# Construction of a Cerebral Hemorrhage Test System Operated in Real-time

**DOI:** 10.1038/srep42842

**Published:** 2017-02-16

**Authors:** Gen Li, Jian Sun, Ke Ma, Qingguang Yan, Xiaolin Zheng, Mingxin Qin, Gui Jin, Xu Ning, Wei Zhuang, Hua Feng, Shiyuwei Huang

**Affiliations:** 1 College of Bioengineering, Chongqing University, Chongqing, China; 2 College of Biomedical Engineering, Third Military Medical University, Chongqing, China; 3 Department of Neurosurgery, Southwest Hospital, Chongqing, China; 4 Research Center of Biomedical Engineering, Chongqing University of Posts and Telecommunications, Chongqing, China

## Abstract

The real-time monitoring and evaluation of the severity and progression of cerebral hemorrhage is essential to its intensive care and its successful emergency treatment. Based on magnetic induction phase shift technology combined with a PCI data acquisition system and LabVIEW software, this study established a real-time monitoring system for cerebral hemorrhage. To test and evaluate the performance of the system, the authors performed resolution conductivity experiments, salted water simulation experiments and cerebral hemorrhage experiments in rabbits and found that when the conductivity difference was 0.73 S/m, the phase difference was 13.196°. The phase difference change value was positively proportional to the volume of saline water, and the conductivity value was positively related to the phase difference of liquid under the same volume conditions. After injecting 3 mL blood into six rabbits, the average change in the blood phase difference was −2.03783 ± 0.22505°, and it was positively proportional to the volume of blood, which was consistent with the theoretical results. The results show that the system can monitor the progressive development of cerebral hemorrhage in real-time and has the advantages of low cost, small size, high phase accuracy, and good clinical application potentiality.

Cerebral hemorrhage (CH), which is the most lethal danger to human health, refers to the primary brain parenchymal internal hemorrhage. With a very high incidence, CH mostly attacks people above 50 years old. The incidence rate is approximately 60 per 100,000 people, but it is rising. The major causes of CH include hypertension, cerebrovascular atherosclerosis, and cerebrovascular malformation^1^,^2^,^3^. The manifestations include an acute onset, extreme danger, and very high disability and fatality rates. Thus, the early identification and detection of CH are extremely important. The clinical methods commonly used for the detection of CH include angiography, computed tomography (CT) scanning, the cerebrospinal fluid (CSF) method, and magnetic resonance imaging (MRI)^4^,^5^,^6^,^7^.

Angiography requires the injection of a contrast agent into blood vessels, and its long detection time and complex operations make it unpractical for clinical use. Skull CT, which is the most widely used CH detection method, can clearly show the bleeding site, amount of blood released, and the shape of the hematoma, but it cannot detect early CH or provide continuous monitoring. The CSF detection method is generally avoided in clinical use because a lumbar puncture will very likely induce cerebral hernia in CH patients. MRI can help discover structural abnormalities and clarify the cause of CH, but it is less effective and more expensive for the diagnosis of acute CH compared with CT. Currently, no equipment is capable of continuous and real-time detection in the clinic. Due to these limitations in current CH detection methods, we should develop real-time, convenient and noninvasive CH detection equipment.

Magnetic inductive phase shift (MIPS) is a new technique for the detection of lesions in brain tissues (e.g., brain edema), CH, and cerebral ischemia^8^,^9^,^10^. The phase shift in the magnetic induction at a specific frequency, and thereby the tissue lesions, can be detected easily with MIPS. Electrical impedance tomography (EIT) is limited in actual applications^11^, because the surface contact resistance of the electrodes and the high resistivity of the skull result in the attenuation of the injected current and thus severely impact the measurement precision. In comparison, MIPS is a noncontact method and overcomes the effects of the electrode-skin contact impedance and the high resistivity of the skull that are challenges faced by EIT. Thus, MIPS is greatly superior in CH detection. The principle of CH detection by MIPS is similar to that of the detection of brain edema^8^: when an excitation magnetic field (EMF) passes through the target, an induced magnetic field (IMF) will be generated in the target, which changes the original EMF. The changes can be detected by a detection coil and transformed into a group of phase differences between the detection coil voltage and the reference voltage, thereby providing information about a target’s conductivity. The progress of CH will change the electromagnetic properties in the brain, and thus it can be real-time monitored by the continuous measurement of changes in the MIPS between the induction signal and detection signal.

A review^12^ shows that the change in MIPS is positively correlated with the volume of the CH, which is the basis for this detection system. In this work, a self-made coil module and a PCI data collection system were combined with LabVIEW to build a real-time CH detection system. The performance of this system was tested and assessed via a conductivity resolution experiment, salt water simulation experiment, and animal experiment.

## Materials and Methods

### Experimental system

This detection system consisted of four modules: a signal generator, excitation and induction coils, a PCI data collection system, and LabVIEW 2012.

### Signal source

A Tektronix signal generator (AFG3252, America) was used to produce two channels of sinusoid signals with a common frequency and consistent initial phase: one induction signal and one reference signal. The output frequency and power from the signal generator can be regulated. The range of output power from the generator was 2–3 mW. The frequency stability was on the order of 10^−4^, and the signal-to-noise ratio (SNR) in the excitation generator was 30–60 dB. All of these conditions satisfy the requirements for phase precision. The excitation signal was set as 7.7 MHz, 5 Vpp, and the reference signal was set as 7.7 MHz, 100 mVpp.

### Coil model

The coil model was composed of an excitation coil and a detection coil. Both coils were wound in 10 circles by copper-painted covered wires (wire diameter 1 mm) that were closely arranged and well insulated. The coil radius was R = 5.2 cm, and the space of the coaxial placement was 10 cm^13^. The coils were fixated with plastic.

### PCI data acquisition system

PCI is a data acquisition system established by the National Instruments Company (NI Company) that is widely used in various types of data acquisition applications. This system uses a dual-channel high-speed data acquisition card (NI PCI 5124) with a maximum real-time sampling rate of 200 MS/s, 12-bit resolution, 150 MHz of bandwidth, and 8 MB of onboard memory. According to the sampling theorem, a signal below 100 MHz can be sampled directly, so using the acquisition card does not require band-pass sampling for the input signal. In addition, the acquisition card has a 50 dB preamplifier function and can amplify a signal of low amplitude to aid in phase detection and other processing for the late-stage software.

### LabVIEW software platform

The software LabVIEW2012 is used for programming. The sampling rate of the PCI-5124 acquisition card is set as 100 MHz by the software platform, the number of sampling points is set as 400000, and the data obtained by the acquisition card are used to display the results of software phase detection. The phase detection uses the FFT algorithm method, which has the advantages of rapid speed and high precision. Moreover, it can adjust the software platform parameters, making it easier to improve the system performance.

### Detection methods

The MIPS results are expressed as phase difference changes


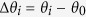


where *θ*_*i*_ is the *i*-th phase difference obtained from the experiments, *θ*_0_ is the original phase difference, and Δ*θ*_*i*_ is the change between them. The change in the phase difference Δ*θ*_*i*_ reflects the severity of the CH^14^.

### Conductivity resolution experiment

The human brain has a complex composition and has a low overall conductivity^15^, so this study aims to determine the conductivity resolution of the system to determine its sensitivity within the variation range of human brain tissue conductivity. A plastic container is fixed on a polyester foam cube (10 cm × 8 cm × 5 cm) at the center between the excitation coil and the detection coil, and each container is filled with 10 mL of one of four differently conductive liquids^16^,^17^: simulated edema fluid, simulated cerebral hemorrhage solution, physiological saline and high-concentration saline (5%). Each liquid is measured five times, and the average value is taken.

### Design of salt water simulation experiment

This experiment simulates the production process of cerebral hemorrhage animal models and also studies the MIPS change caused by changes in the closed chamber volume. A syringe pump is used to inject the same four different conductivity liquids (simulated edema fluid, simulated cerebral hemorrhage solution, physiological saline and high-concentration saline (5%)) into the above-described plastic containers at a rate of 1 mL/min. Each liquid is measured 5 times, and the average data value is obtained.

### Design of animal CH experiment

**Figure 1:**
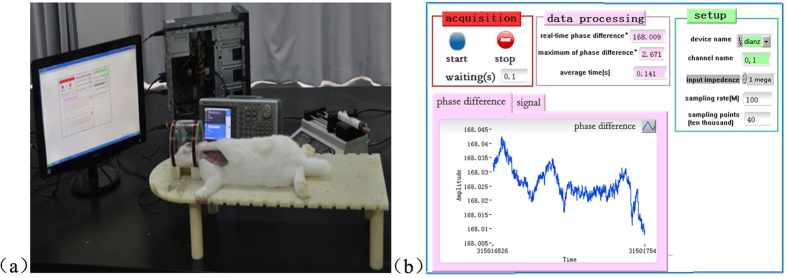
(**a**) Monitoring system of rabbit cerebral hemorrhage experiment. (**b**) The software platform.

All animal experiments were performed in accordance with the guidelines from the Administration of Animal Experiments for Medical Research Purposes issued by the Ministry of Health of China. The protocol used was reviewed and approved by the Animal Experiments and Ethical Committee of Third Military Medical University (TMMU, Chongqing, China). All efforts were made to minimize the suffering of rabbits during experiments. Eleven rabbits (2.1–2.5 kg) were obtained from Daping Hospital, Chongqing, China. They were divided into the experimental group (n = 6) and control group (n = 5). For the experimental group, after anesthesia via the ear vein (25% urethane, 5 mL/kg), 6 mL of blood was collected from the heart^18^. Then, autologous blood and 5% heparin were mixed at a ratio of 2:1. Bleeding in the internal capsule was simulated via a stereotactic approach^19^. The autologous blood was injected via an injection pump into the inner capsule to artificially induce CH. With the cross suture in the rabbit brain as the base point, the injection point in the inner capsule was located 6 mm to the right of the coronal suture and 1 mm parallel to the sagittal suture. The rabbit was kept inactive throughout the experiments (except for basic physiological activities such as breathing and heartbeat). The injection rate of the pump was set at 0.33 mL/h, the injection volume was 3 mL, and the injection time was 9 min. Rabbits in the control group received the same procedure without a blood injection. The software and hardware of the experimental system are shown in Fig. 1(a,b).

### MIPS data analysis

Because factors such as power frequency interference and cardiopulmonary activity interference during the measurement process add noise to the MIPS signal and exhibitnon-stationary characteristics, we preprocessed the MIPS signal using a wavelet transform. First, the Daubechies wavelet (4th order) was used to decompose the MIPS signal into a 10-layer wavelet. The rabbit breathing frequency was below 6 Hz, and the heart and lung activity interference signal components were mainly concentrated in D1~D8. Therefore, we removed the components of the D1~D8 layer, restored the sequence of the D9~D10 layer, performed wavelet reconstruction, and then used the wavelet transform for threshold de-noising. Moreover, the initial phase of the ten rabbits was set to zero, which enables more intuitive observation of the MIPS absolute value change caused by blood injection after the elimination of breathing during the experimental process.

### MRI analysis

Magnetic resonance imaging methods were used to obtain the cerebrospinal fluid distribution image with the increased blood injection volume. A 3.0 T Magnetic Resonance Imager (Magnetom Spectra with A Tim + Dot System, Siemens) was used to scan with T2 weighted three-dimensional (3D) variable flip angle TSE (SPACE) sequence. The scanning plane was perpendicular to the body, and the scanning parameters were set as follows: TR is 1300 ms; TE is 44 ms, ETL is 49; FOV is 160 mm × 160 mm; matrix is 320 mm × 275 mm; scanning slice is set to 0.5 mm; and the number of slices is 192. The 3D SPACE sequence employs variable low flip angle refocusing RF pulses which can achieve a long echo train length and clinical acceptable acquisition time. As the T2 value of CSF is much longer than surrounding tissue, the CSF signal decays much slower and demonstrates a brighter signal compared to surrounding tissue using an echo time of 44 ms. Image processing was performed using Amira 5.4.3 software (Visage Imaging, Australia).

### Statistical analysis

All of the data are expressed as the mean ± standard deviation from 5 independent experiments. The salt water simulation data were analyzed with a paired-samples t-test. The rabbit CH experimental data were analyzed with a bilaterally independent t-test. Statistical analyses were performed using SPSS software version 19.0 (SPSS Inc., Chicago, USA).

## Results

### Conductivity resolution experiment

The conductivity resolution results are shown in Table 1. The higher the overall conductivity of an object is, the more sensitive a detection system can be. In view of the fact that the conductivity difference between the cerebral hemorrhage and simulated cerebral edema fluids was only 0.73 S/m, the phase difference was 13.196°, indicating that the system could detect small conductivity changes in the detected objects.

**Figure 2:**
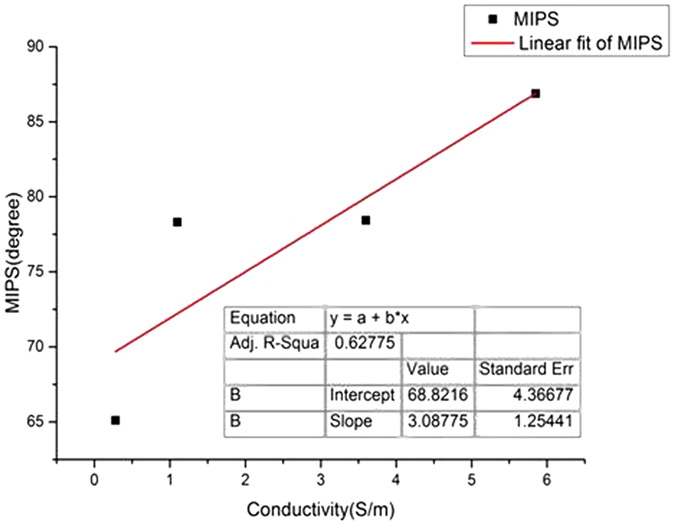
Conductivity-phase characteristic fitting curve.

The linear fit curve between the conductivity (P value < 0.05) obtained by fitting the data in the table using Origin 8.0 and the MIPS is shown in Fig. 2. The overall trend of the linear fit curves for the relationship between conductivity and MIPS indicated an approximately proportional relationship between conductivity and MIPS. The results also suggested that in the animal model experiment, the whole intracranial electrical conductivity was decreased with increased blood injection volume; therefore, the MIPS value should decrease monotonically. The experiment provides a reference for the following animal experiments.

### Salt water simulation experiment

**Figure 3:**
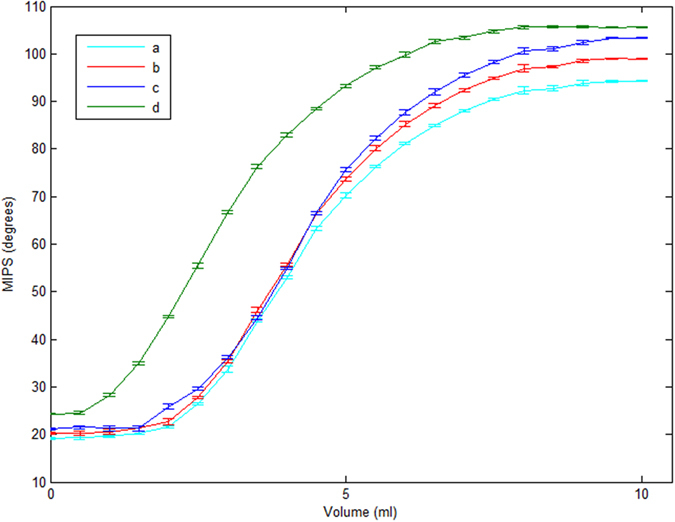
MIPS changes in 4 different liquid injection processes. (**a**) simulated edema fluid; (**b**) simulated cerebral hemorrhage solution; (**c**) physiological saline; (**d**) high-concentration saline (5%).

All of the measurement results were re-sampled to obtain a total of 20 sets of four different conductivity solutions (5 groups for each of the 4 different conductivity solutions) containing 21 discrete variables. Then, the 5 sets of data from the four different conductivity solutions were averaged. The mean MIPS changes for the four different liquids are shown in Fig. 3, and all of them exhibited an upward trend during the injection process. The MIPS change rate is faster during the first 5 mL injection period than during the subsequent 5 mL injection period. This difference is due to the following two reasons: (1) the liquid container has an inverted cone shape, with a small radius on the bottom and a large radius on top, causing the liquid level to rise faster initially, then more slowly and (2) the volume changes lead to changes in the measurement point position of the detection coil, and the liquid overflow from the most sensitive areas decreases the sensitivity of the liquid during the injection process.

The MIPS values after injection of 5 mL and 10 mL of the four different conductivity solutions are shown in Table 2. The conclusion from the conductivity-resolution experiments is further supported by the results that liquids with larger conductivities exhibit a larger phase change for the same volume change. The MIPS value after injection of 5 mL and 10 mL of the four different conductivity solutions were compared using the paired sample t-test (α = 0.05), and a P value less than 0.05 was obtained. This finding indicates that there is a significant difference in the MIPS values between 5 mL and 10 mL solutions with the same conductivity. Hence, it can be concluded that when solution conductivity is constant, the MIPS value changes with the volume of the solution.

### Rabbit CH experiment

**Figure 4:**
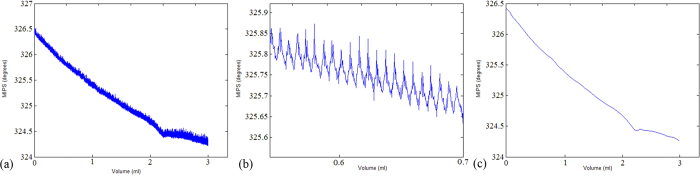
(**a**) Original MIPS data for rabbit No. 1. (**b**) Original MIPS data for rabbit No. 1 from 0.6 mL to 0.7 mL. (**c**) MIPS data after signal preprocessing for rabbit No. 1.

Figure 4(a) shows the original MIPS change trends of the blood injection process for rabbit No. 1. The MIPS declined with the increasing blood injection volume, with a relatively stable declining curve, falling by a total of 1.9676 degrees. After injecting 1 mL, 2 mL, and 3 mL of blood, the measured MIPS changes were 0.72638°, 0.63758°, and 0.5627°, respectively. The MIPS changes exhibited only minor fluctuations in a local region, with a volatility of approximately 0.035°. Amplification of the MIPS data from rabbit No. 1 in the 0.6 mL to 0.7 mL observation interval is shown in Fig. 4(b). The interference from lung activity is mixed. As shown in Fig. 4(c), the original signal from rabbit No. 1 becomes very smooth after noise reduction and filtering are applied. Figure 4(c) indicates that with the increase in blood volume, the change in the MIPS value decreases. In the salt water simulation experiment, MIPS was obtained by subtracting the phase before the injection from the phase during detection. In the rabbit CH experiment, MIPS was obtained by subtracting the detection phase from the phase before the injection. Therefore, the MIPS trend for the rabbit CH experiment was different from the salt water simulation experiment.

**Figure 5:**
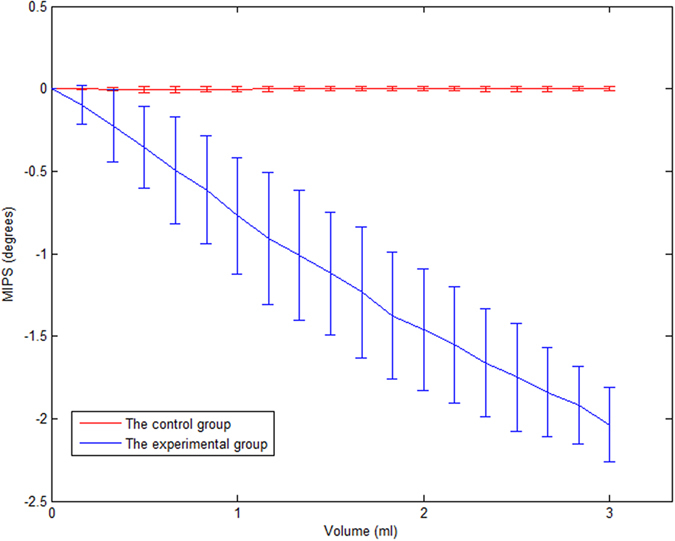
Mean MIPS value and standard deviation for 6 experimental rabbits and 5 control rabbits.

Figure 5 shows the mean measured MIPS value and standard deviation of 6 experimental rabbits and 5 control rabbits with increasing blood injection volumes. For the experimental group, after injecting the first 1 mL of blood, the average MIPS value declined by 0.77065°; after injecting the second 1 mL of blood, the average MIPS value declined by 0.69082°; and after injecting the third 1 mL of blood, the average MIPS value declined by 0.57636°. The control group did not receive injected blood, and only minor cranial bleeding was observed in the damaged cranial bone. The cerebrospinal fluid with the highest intracranial electrical conductivity was basically in a normal cycle; therefore, the small bleeding volume and low conductivity caused weak MIPS changes. The MIPS results from the experimental rabbits and control rabbits were analyzed with bilaterally independent t-tests, and P values less than 0.05 were obtained, indicating significant differences in the MIPS between the experimental group and control group. The results show that the MIPS change was mitigated by the increasing injected blood volume.

### MR image

**Figure 6:**
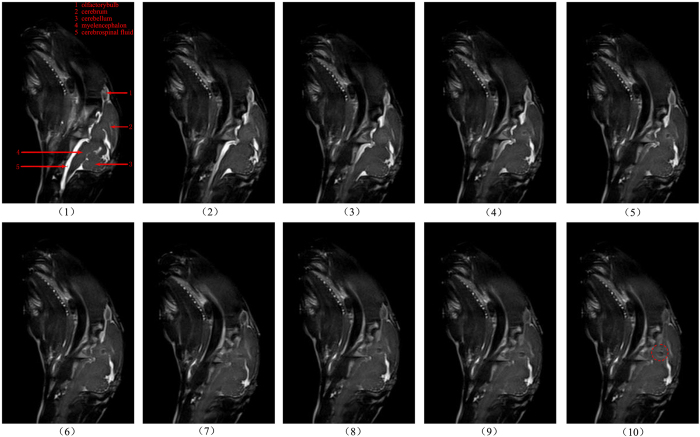
MR image of the 98th slices of the sagittal plane for the experimental group rabbits with the increase in the blood volume injection. From (1) to (10), the injected blood volumes are 0, 0.33 mL, 0.66 mL …to 3 mL. The red circle in (10) shows the injected blood.

Figure 6 is an MRI image verifying cerebral hemorrhage in the experimental group rabbits. Researchers selected the 98th slice (98/192) as the sagittal plane of the image; the lower portion of the figure is an MR image acquired every 0.33 mL. The total volume of the blood injection was 0 to 3 mL.

As shown in Fig. 6, the white highlighted portion is the volume of cerebral spinal fluid. With the continuous injection of autologous arterial blood, the white highlighted area decreased as the cerebrospinal fluid was continuously discharged.

## Discussion

### Conductivity resolution experiment

MIPS is based on the principle of phase changes during measurements and changes in the overall conductivity of biological tissue^20^. Compared to normal circumstances, cerebral hemorrhage causes an overall intracranial electrical conductivity change, which leads to a MIPS change. Therefore, it is theoretically feasible to detect cerebral hemorrhage by MIPS^21^.

The phase difference formula 
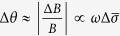
 derived by Griffiths *et al*. was reported in 1999^22^. In the formula, Δ*θ* is the overall average brain phase difference, is Δ*B* the magnetic induction intensity generated by the brain, *B* is the magnetic field strength generated by the excitation coil, *ω* is the excitation signal angular frequency, and 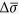 is the brain’s overall average conductivity change. The formula shows that when the excitation signal frequency is constant, the MIPS size is proportional to the conductivity of the measured target. The experimental results are consistent with the above theory and further verify the feasibility of the system to detect cerebral hemorrhage.

### Salt water simulation experiment

The established animal model method used was to perform craniotomy of the rabbits’ skulls, which involves embedding the thin tube into the parenchyma of the skull and then injecting blood slowly with a syringe pump. Therefore, this experimental model simulates the process of intracerebral hemorrhage. The results show that the overall MIPS value changes quickly at first and then slowly, indicating that the location of the measured target will affect the sensitivity of the system^12^. In future experiments, we should control the location of the blood injection point and try to maintain it in a region with large detection coil sensitivity to improve MIPS detection sensitivity.

### Rabbit CH experiment

The time of the single phase difference obtained by the system is 0.2368 s, which could meet the requirements of real-time monitoring. The accuracy of the phase difference differentiation could also meet the monitoring needs^23^. Thus, the system can accurately monitor the progress of cerebral hemorrhage in real-time.

As reported^24^,^25^, the contents of the cranial cavity are composed of brain tissues, CSF, and cerebral blood flow (CBF), with the conductivity varying in the order of CSF > CBF > brain tissues. At the early stage of CH, the conductivity changes rapidly due to the CSF-induced compensatory action. Then, the changes slow down due to the compensatory action from the blood. After the compensatory action disappears, the conductivity changes more slowly; thus, the MIPS theoretically changes in the same way as the conductivity^22^,^26^. However, inconsistency exists between the experimental results and the theoretical analysis above. We believe that CH is a very complex process, and the CH-induced conductivity changes are not only caused by compositional changes, but also by extrusive denaturation at the bleeding sites^27^. Moreover, in our experiments, we used an artificially induced acute cerebral hemorrhage using a surgery that might cause spontaneous intracalvarial hemorrhage, which is largely different from a real CH^28^. Moreover, owing to the limitation in time, only a small sample size was tested. In the future, more experiments are needed to account for the differences between experiments and theories. Nevertheless, however the conductivity changes, its trend is obvious. Our system can precisely monitor the changes and thereby monitor the progress of CH in real time. In addition, MIPS might also be applied to monitoring atrial fibrillation (AF)^29^.

## Conclusions

The salt water simulation experiments and animal experiments show that the newly built magnetic induction detection system can detect the real-time progress of cerebral hemorrhage. The advantages of low cost, high precision and high sensitivity endow this system with great application prospects.

To improve the systemic performance, a reference coil or shielding materials will be added to shield the coil and eliminate electromagnetic interference. A higher-resolution data collection board can be used to acquire more precise MIPS. As reported, a 16-bit collection board^30^ achieves a precision of 0.001°. Moreover, new or better system parameters can be selected. To optimize the rabbit experiments, the sample volume of the rabbit cerebral hemorrhage will be enlarged. Cerebral hemorrhages induced at different sites will also be studied.

## Additional Information

**How to cite this article**: Li, G. *et al*. Construction of a Cerebral Hemorrhage Test System Operated in Real-time. *Sci. Rep.* **7**, 42842; doi: 10.1038/srep42842 (2017).

**Publisher's note:** Springer Nature remains neutral with regard to jurisdictional claims in published maps and institutional affiliations.

## Figures and Tables

**Table 1:** Phase difference measurement results of different liquids.

Target solution	Edema	Hemorrhage	Normal saline	Saline (5%)
Conductivity (S/m)	0.281	1.101	3.6	5.85
Phase difference (°)	65.108	78.304	78.438	86.883

**Table 2:** MIPS values after injecting 5 mL and 10 mL of 4 solutions of different conductivities.

Target solution	MIPS (°)	
	5 mL	10 mL
Edema	70.2040 ± 0.5360	94.2350 ± 0.1104
Hemorrhage	73.7142 ± 0.3616	98.9468 ± 0.1229
Normal saline	75.6464 ± 0.3994	103.3409 ± 0.1248
Saline (5%)	93.2278 ± 0.2534	105.6064 ± 0.1006
